# Supplementary material for: A Delphi consensus on the management of oral anticoagulation in patients with non-valvular atrial fibrillation in Spain: ACOPREFERENCE study
Source: PLoS One. 2020 Jun 1;15(6):e0231565. doi: 10.1371/journal.pone.0231565 (PMC7263623; doi:10.1371/journal.pone.0231565)
Supplement: S1 Appendix — (DOCX) [file pone.0231565.s001.docx]

**Table 1.** **Evaluation of thromboembolic and bleeding risk for treatment decision**

| **Items** |  |  |
| --- | --- | --- |
| 1. The primary reason for initiating anticoagulant treatment is to reduce the risk of stroke | | |
| 2. The treatment decision should be individualised based on the evaluation of the thromboembolic and bleeding risk of each patient | | |
| 3. The most appropriate scale to evaluate the thromboembolic risk of patients with NVAF who are candidates for oral anticoagulation treatment is CHADS_2_ | | |
| 4. The most appropriate scale to evaluate the thromboembolic risk of patients with NVAF who are candidates for oral anticoagulation treatment is CHA_2_DS_2_-VASc | | |
| 5. The CHA_2_DS_2_-VASc scale has a greater discriminative capacity for identifying patients with lower thromboembolic risk | | |
| 6. Of the criteria considered in the CHA_2_DS_2_-VASc scale, age is the thromboembolic risk factor with the greatest weight | | |
| 7. Of the criteria considered in the CHA_2_DS_2_-VASc scale, stroke is the thromboembolic risk factor with the greatest weight | | |
| 8. Anticoagulant treatment is recommended for prevention of thromboembolism in male patients with CHA_2_DS_2_-VASc ≥2 | | |
| 9. Anticoagulant treatment is recommended for prevention of thromboembolism in female patients with CHA_2_DS_2_-VASc ≥3 | | |
| 10. Initiation of anticoagulant therapy should be considered in patients with a CHA_2_DS_2_-VASc score of 2 in female patients and 1 in male patients after assessing the risk-benefit ratio and the patient’s preference | | |
| 11. The use of bleeding risk assessment scales should be considered in patients with NVAF treated with oral anticoagulants to identify modifiable major risk factors | | |
| In patients with NVAF, bleeding risk should be assessed using: | | |
| 12. The HAS-BLED scale  13. The ATRIA scale  14. The HEMORR_2_HAGES  15. The ORBIT | | |
| 16. A patient at high bleeding risk is considered as one having a HAS-BLED score ≥3. | | |
| 17. The SAMe-TT_2_R_2_ scale should be used to predict poor INR control in candidates for oral anticoagulation when deciding treatment. | | |
| 18. The SAMe-TT_2_R_2_ scale is routinely used for patient management | | |

NVAF: non-valvular atrial fibrillation

**Table 2. Choice of anticoagulant treatment in patients with NVAF**

| **Items** |  |  |
| --- | --- | --- |
| 1. In general, treatment with VKAs should be the initial anticoagulant therapy in patients with NVAF in whom anticoagulation is indicated. | | |
| 2. In general, treatment with DOACs should be the initial anticoagulant therapy in patients with NVAF in whom anticoagulation is indicated. | | |
| 3. DOACs should be a treatment option in patients with NVAF in whom INR control is suboptimal with VKAs in spite of good treatment adherence or if VKAs are contraindicated. | | |
| 4. In patients on treatment with VKAs who have good INR control, switching to a DOAC is not recommended except in the case of ischaemic stroke with clinical and neuroimaging criteria for high risk of intracranial haemorrhage (ICH), severe arterial thromboembolic episodes, or inability to access INR controls. | | |
| DOACs are a cost-effective alternative to classic oral anticoagulants in: | | |
| 5. All patients with NVAF. | | |
| 6. Specific profiles of patients with NVAF. | | |
| 7. Patients at high thromboembolic risk. | | |
| 8. The use of DOACs could be a more recommendable option in patients at higher risk of thromboembolic or bleeding complications, and for patients who have frequent poor control of anticoagulation with VKAs. | | |
| 9. The use of VKAs remains significantly more predominant than DOACs in patients with NVAF in Spain. | | |
| The use of DOACs would be a suitable option in patients with NVAF who are candidates for anticoagulant therapy in the following situations (items 10-14): | | |
| 10. Known hypersensitivity or specific contraindication to the use of acenocoumarol or warfarin. | | |
| 11. History of ICH if it is assessed that the benefits of anticoagulation outweigh the risk of bleeding. | | |
| 12. Patients with ischaemic stroke who meet clinical and neuroimaging criteria for high risk of ICH, defined as the combination of HAS-BLED ≥3 and grade III-IV leukoaraiosis and/or multiple cortical microbleeds | | |
| 13. Patients on treatment with VKAs who suffer severe arterial thromboembolic episodes despite having good INR control | | |
| 14. Patients who have started treatment with VKAs in whom INR control cannot be maintained within the therapeutic range (2–3) despite good compliance, considering that INR control is inadequate when TTR calculated by the Rosendaal method is <65% when this method is available or the percentage of INR values within the therapeutic range (direct TRT) is <60%, considering an assessment period of 6 months in both cases, excluding INR values of the first month or periods of change resulting in modification of the VKA regimen. | | |
| 15. Patients who have failed to achieve an optimal therapeutic range within 3 months from the start of VKA treatment. | | |
| 16. Patients in whom access to conventional INR control is not possible. | | |
| To initiate treatment with DOACs, patients with NVAF and indication for anticoagulant therapy should meet the following criteria (items 17–21): | | |
| 17. No specific contraindications for DOACs | | |
| 18. At least one of the previously mentioned clinical situations (items 10-14) | | |
| 19. History of previous good treatment compliance | | |
| 20. Ability of the patient to understand the risk-benefit ratio of anticoagulation and/or family/social care who understand it | | |
| 21. Reliable possibility of periodic follow-up of the necessary assessments (clinical examinations, renal function monitoring) | | |
| 22. All the criteria stated in the TPR must be met before DOACs can be prescribed to a patient with NVAF who is a candidate for anticoagulant therapy. | | |
| 23. There are clinical situations not identified in the TPR where the benefit of DOACs could be superior to VKAs. | | |
| 24. In patients with NVAF, the risk of severe bleeding is lower with the use of DOACs than with VKAs. | | |
| 25. The bleeding with greatest risk for this patient is ICH. | | |
| 26. In patients with NVAF, the risk of ICH is lower with the use of DOACs than with VKAs. | | |
| 27. It is reasonable that DOACs are indicated in patients who have a greater risk of ICH. | | |
| 28. Among patients with NVAF, male patients with CHA_2_DS_2_-VASc ≥3 and female patients with CHA_2_DS_2_-VASc ≥4 should be candidates to receive DOACs. | | |
| 29. Patients with high bleeding risk should be candidates to be treated with DOACs. | | |
| 30. Patients with a score greater than 2 on the SAMe-TT_2_R_2_ scale should be candidates to receive initial anticoagulant therapy with DOACs. | | |
| 31. DOACs are a suitable alternative for polymedicated patients. | | |
| 32. Assessment of renal function is essential when prescribing DOACs to a patient with NVAF. | | |
| 33. During treatment with DOACs, renal function should be assessed at least once a year, particularly in clinical situations where there is a greater risk of renal function deterioration. | | |
| 34. The efficacy and safety profile of each DOAC should be taken into account when prescribing a DOAC. | | |
| 35. When prescribing a DOAC, it should be taken into account whether a specific reversal agent for the DOAC considered is available. | | |
| 36. It is essential to assess the expected degree of treatment adherence before initiating treatment with DOACs. | | |
| 37. Patients treated with DOACs could have lower treatment adherence. | | |
| 38. Proper patient education should be ensured by an adequate explanation of the disease and the benefits and risks of anticoagulant therapy. | | |
| 39. After explaining to the patient the different options for anticoagulant treatment (VKAs and DOACs), the patient’s preference should be taken into account when prescribing. | | |
| DOAC: Direct-acting oral anticoagulant; INR: International normalised ratio; NVAF: non-valvular atrial fibrillation: TPR: Therapeutic Positioning Report; VKA: Vitamin K antagonist | | |

**Table 3. Participation and education of the anticoagulated patient**

| **Items** |  |  |
| --- | --- | --- |
| 1. Patients often have little knowledge about the available anticoagulant treatments. | | |
| 2. A substantial proportion of patients is unaware of DOACs. | | |
| 3. Patients who are candidates for anticoagulation usually have doubts about the implications of being on anticoagulant treatments. | | |
| 4. Before initiating anticoagulant treatment, the patient should be educated about the responsibility involved in the use of this treatment. | | |
| 5. The patient is provided with written as well oral information about the anticoagulant treatment on a routine basis in the physician’s office. | | |
| 6. Patient education is essential to foster in the anticoagulated patient a responsible attitude to their disease and their treatment. | | |
| 7. Patients should be instructed about the need to inform that they are receiving anticoagulation in acute or emergency situations. | | |
| 8. Patient should be specifically informed about whether there is a reversal agent for their treatment. | | |
| Education of the anticoagulated patient is a relevant tool for (items 9–14): | | |
| 9. Achieving optimum control of anticoagulation. | | |
| 10. Reducing thromboembolic and bleeding risk. | | |
| 11. Reducing the risk of complications from oral anticoagulant treatment. | | |
| 12. Achieving good treatment adherence. | | |
| 13. Encouraging a lifestyle appropriate to their condition. | | |
| 14. Reducing patient visits to the hospital. | | |
| Education of the anticoagulated patient should address (items 15–32): | | |
| 15. The reason for anticoagulation. | | |
| 16. The risk of stroke and how to detect it. | | |
| 17. Information about the anticoagulant drugs available and suitable for their case. | | |
| 18. The risk-benefit ratio of anticoagulation. | | |
| 19. The dosing of anticoagulant drugs (adequate explanation about how to take them). | | |
| 20. The need or not for anticoagulation monitoring. | | |
| 21. Action to be taken in case of missed doses. | | |
| 22. Possible drug interactions with the treatment. | | |
| 23. Possible adverse effects of the treatment. | | |
| 24. The risk of excessive or insufficient anticoagulation. | | |
| 25. Recommended diet and social habits/lifestyle. | | |
| 26. Action to be taken in case of side effects. | | |
| 27. When to discontinue anticoagulant treatment (elective surgery, dental procedure, etc.). | | |
| 28. How to stop and restart anticoagulant treatment in specific situations in which anticoagulant treatment should be interrupted (elective surgery, dental procedure, etc.). | | |
| 29. When patients should contact their physician. | | |
| 30. Availability of a specific reversal agent for their treatment. | | |
| 31. The mechanisms for reversing the patient’s anticoagulation. | | |
| 32. The importance of treatment adherence. | | |
| 33. In general, the time spent on patient education in the office is insufficient. | | |
| 34. The physician does not always have sufficient adequate patient education materials on hand in the office. | | |
| 35. Active and responsible participation of the physicians treating the anticoagulated patients is essential for patient education. | | |
| 36. The role of nursing staff is essential for the education of anticoagulated patients. | | |
| 37. The information on the disease and anticoagulant treatment should be reinforced in the routine follow-up visits of patients with NVAF. | | |
| 38. Physician education through attendance of courses, workshops and publications related to anticoagulant therapy is essential for the health education of anticoagulated patients. | | |
| 39. It is recommendable for patients, supported by healthcare professionals (physicians and nursing staff), to participate in the management of their disease through health education programs, patient education forums and educational activities (courses, workshops, conferences, etc.). | | |
| 40. New technologies (computer applications, multimedia educational programs. etc.) offer useful tools to facilitate and promote the education of anticoagulated patients. | | |
| 41. Patient associations (e.g., Association of Anticoagulated Patients) have a key role in the health education of anticoagulated patients. | | |
| 42. Shared decisions can ensure that medical care respects the patient’s needs, values and preferences. | | |

DOAC: Direct-acting oral anticoagulant; NVAF: non-valvular atrial fibrillation

**Table 4. Use of anticoagulants in specific cardiology situations**

| **Items** |  |  |
| --- | --- | --- |
| 1. Patients with AF who are to undergo electrical cardioversion should receive anticoagulation for at least 3 weeks prior to cardioversion. | | |
| 2. In patients at high risk of stroke, long-term anticoagulant therapy should be continued after cardioversion according to the recommendations for long-term anticoagulation, regardless of the method of cardioversion or maintenance of sinus rhythm. | | |
| 3. In patients without risk factors for stroke, maintenance of anticoagulation for 4 weeks after cardioversion is recommended. | | |
| 4. Treatment with VKAs is the therapy of choice in patients who are to undergo cardioversion. | | |
| 5. Treatment with DOACs is the therapy of choice in patients who are to undergo cardioversion. | | |
| 6. The lack of control of VKAs makes their use difficult prior to cardioversion. | | |
| 7. It is reasonable to use oral anticoagulation (DOAC or VKA) for at least 8 weeks after surgical or catheter ablation in low risk patients. | | |
| 8. In patients at high risk of stroke undergoing surgical or catheter ablation of atrial fibrillation, regardless of its success, anticoagulation should be maintained indefinitely. | | |
| 9. In patients at high risk of stroke undergoing surgical left atrial appendage occlusion or exclusion, it is recommended to continue with oral anticoagulation. | | |
| 10. In patients with NVAF at high risk of stroke who have undergone elective coronary stent implantation for stable coronary disease, triple therapy with aspirin, clopidogrel and oral anticoagulation for 1 month may be considered, and the bleeding risk of the patient assessed subsequently. | | |
| 11. After an ACS with stent implantation in patients with NVAF and risk of stroke, triple therapy with aspirin, clopidogrel and OAC should be considered for 1-6 months depending on the bleeding risk. | | |
| 12. From 1 year after the ACS, oral anticoagulation should be maintained without antiplatelet agents. | | |

ACS: acute coronary syndrome; AF: atrial fibrillation; DOAC: Direct-acting oral anticoagulant; NVAF: non-valvular atrial fibrillation: VKA: Vitamin K antagonist
