# Supplementary material for: A Delphi consensus on the management of oral anticoagulation in patients with non-valvular atrial fibrillation in Spain: ACOPREFERENCE study
Source: PLoS One. 2020 Jun 1;15(6):e0231565. doi: 10.1371/journal.pone.0231565 (PMC7263623; doi:10.1371/journal.pone.0231565)
Supplement: S2 Appendix — (DOCX) [file pone.0231565.s002.docx]

| **Delphi panel experts** | **Site** |
| --- | --- |
| Alain Laskibar Asua | Hospital Universitario de Basurto |
| Alejandro Rodríguez Vilela | Hospital Arquitecto Marcide |
| Alejandro Villanueva Afán de Ribera | Hospital Rey Juan Carlos |
| Alicia Ibáñez Criado | Hospital General de Alicante |
| Amparo Benedicto | Hospital Universitario de la Princesa |
| Ana Ayesta López | Hospital del Sureste |
| Andrés Raúl May | Hospital la inmaculada |
| Antonio Agarrado Luna | Hospital SAS Jerez |
| Antonio Enrique Gómez Menchero | Hospital Juan Ramon Jiménez |
| Antonio Jesús Martín de la Higuera | Hospital Virgen De Las Nieves |
| Antonio Luis Gámez López | Hospital San Juan de la Cruz-Úbeda, Jaén |
| Antonio Tovar Martínez | Hospital Morales Meseguer |
| Antonio Ortiz Carrellán | Hospital Virgen del Rocío |
| Beatriz Mascarell Gregori | Hospital LLuis Alcanyís |
| Beatriz Moreno Djadou | Hospital San Pedro |
| Belén Mari López | CHUC |
| Belén Puigdueta Vindel | Hospital Universitari del Tajo |
| Berta Ferreiro Rodríguez | Hospital de Mataró |
| Carlos Alexandre Almeida Fernández | Complejo Asistencial de Ávila |
| Carlos Israel Chamorro Fernández | Hospital Virgen de los Lirios, Alcoy |
| Carmela Alonso Gutiérrez | Hospital Nuestra Señora del Rosario |
| Cristina Goena Vives | Hospital de Mendaro |
| Cristina Victoria Iglesia Carreño | Hospital Povisa |
| Cristóbal Navas Navas | Hospital Esperit Sant |
| Daniel García Fuertes | Hospital Santa Bárbara |
| David Vilades Medel | Hospital Santa Creu i Sant Pau |
| Diana Domingo Valero | Hospital la Fe |
| Diego Fernández Redondo | Complejo Hospitalario Universitario de Pontevedra |
| Diego Lorente Carreño | Hospital San Pedro |
| Eduardo Casas Rojo | Ramon y Cajal |
| Eduardo Sebastián López Sánchez | Hospital La Inmaculada Huercal-Overa Almería |
| Elena Laherran Rodríguez | Hospital Rio Carrión, Palencia |
| Elisa Blanco González | Hospital Álvaro Cunqueiro |
| Eloy Gómez Mariscal | H.U. Infanta Leonor |
| Eloy Rueda Calle |  |
| Esmeralda Capin Sampedro | HUCA |
| Esther Marcos Blanco | Hospital de Móstoles |
| Eva Guillaumet Gasa | Hospital Parc Taulí |
| Eva María Pereira López | H.U. Lucus Augusti |
| Fernando Álvaro López Sánchez | H Virgen de la Salud |
| Francisco González Vílchez | Hospital Marqués de Valdecilla (Santander) |
| Francisco Javier Cuesta Cuesta | Hospital Universitario La Princesa |
| Francisco Javier Merelles Otero | Hospital de Verin |
| Francisco Javier Rivera Rabanal | H.U. Virgen Macarena |
| Francisco José Guerrero Márquez | H. Virgen del Rocío |
| Francisco Manuel Salmerón Martínez | CHU Albacete |
| Francisco Martin Herrero | Hospital Universitario de Salamanca |
| Francisco Carrasco Ávalos | H.U. Reina Sofía, Córdoba |
| Gabriel Retegui García de Quesada | Hospital San Juan Grande |
| Gala Caixal Vila | Hospital Clinic |
| Gonzalo Peña Pérez | Hospital San Rafael |
| Héctor Marrero Santiago | Centro Hospitalario Universitario Insular de Gran Canaria |
| Helen Margarita Valenzuela Leal | Hospital de Figueres |
| Ignacio Alins Rami | Hospital San Jorge, Huesca |
| Irene Rilo Miranda | Hospital Universitario Donostia |
| Isabel María Piñero Uribe | Hospital Carlos Haya |
| Isidoro Adolfo Rodríguez Tejero | Hospital Quirón Infanta Luisa |
| Itsaso Rodríguez Guinea | Hospital Guadalajara |
| Jaime Fernández-Dueñas Fernández | Clónica Ciudad Jardin |
| Javier Chimeno García | Complejo Asistencial de Zamora |
| Javier Fuertes Beneitez | Hospital Universitario La Paz |
| Javier García Pérez-Velasco | Hospital Príncipe de Asturias |
| Javier Jiménez Bello | Hospital General Universitario De Valencia |
| Javier León Jiménez | Complejo Hospitalario Universitario de Huelva |
| Javier López Díaz | Hospital Clínico de Valladolid |
| Javier Vara Manso | Complejo Asistencial Universitario de León |
| Jesús Ignacio Domínguez Calvo | Hospital El Bierzo |
| Joaquín Rueda Soriano | Hospital La Fe |
| Joaquina Belchi Navarro | Hospital General de Valencia |
| Jorge Rodríguez Capitán | Hospital Comarcal de Antequera |
| José Antonio Moro López | Hospital Arnau Vilanova, Valencia |
| José Castillo Ortiz | Hospital de Poniente |
| José Enrique Castillo Lueña | Hospital Miguel Servet |
| José Ferrando Cervello | Hospital Universitario Dr. Peset |
| José Francisco Carretero Ruiz | Hospital Costa del Sol |
| José González Ruiz | Hospital de Galdakao-Usansolo |
| José López Aguilera | Hospital Universitario Reina Sofia |
| José Luis Moriñigo Muñoz | Hospital Universitario de Salamanca |
| José María Basurto Hoyuelos | Hospital Universitario de Basurto |
| José Nieto Tolosa | Hospital de la Vega Lorenzo Guirao |
| José Ramón Balaguer Malfagón | Hospital de la Ribera |
| Juan Carlos Castillo Domínguez | Hospital Reina Sofia |
| Juan Francisco Cueva Recalde | Hospital Clínico Universitario Lozano Blesa |
| Juan Luis Bonilla Palomas | Hospital San Juan de la Cruz, Úbeda |
| Juan Robledo Carmona | Hospital Virgen de la Victoria de Málaga |
| Julia Seller Moya | Hospital de Denia |
| Julio Martínez Flórez | Hospital San Pedro |
| Laura Cejudo Diaz del Campo | Hospital General La Mancha Centro |
| Laura Quintas Ovejero | Hospital de Mendaro |
| Lorenzo Hernando Marrupe | Hospital Universitario Fundación Alcorcón |
| Luis Morcillo Hidalgo | Virgen de la Victoria |
| Luis López González | Hospital Clínica Benidorm |
| M Antonia Busta Vallina | Hospital Valle Nalon |
| Mª Victoria Moreno Flores | Hospital Vega Baja |
| Manuel Anguita Sánchez | Hospital Reina Sofia |
| Manuel Fernando Gonzales Vargas-Machuca | Hospital San Juan de Dios del Aljarafe |
| Manuel Jesús Ruiz Ruiz | Hospital Quirón Salud Campo de Gibraltar |
| María Carmen López Pérez | Clínica Sagrada Familia |
| María Jesús Pinilla Lozano | Centro Sanitario Cinco Villas |
| María José Antolinos Pérez | Hospital Virgen de las Nieves |
| María José Ruiz Olgado | Hospital Virgen de la Concha |
| María Teresa San Agustin Lascorz | Hospital Monteprincipe |
| Mario Missorici Corso | Hospital de Barcelona SCIAS |
| Marta Pombo Jiménez | Hospital Costa del Sol |
| Martín Ruiz Ortiz | Hospital Universitario Reina Sofía |
| Maruan Carlos Chabbar Boudet | H.U. Miguel Servet |
| Mauricio José Pellicer Bañuls | Hospital Clínico Valencia |
| Maximo Migliori | DexeusQuironSalud |
| Mercedes Camprubi Potau | Hospital Joan 23 Tarragona |
| Miguel Angel Ramírez Marrero | Hospital Regional Universitario de Málaga |
| Miguel Angel San Martín Gómez | Hospital de Guadalajara |
| Miguel Angel Simón García | Complejo Hospitalario Universitario de Albacete |
| Miguel Llano Cardenal | H.U.M. Valdecilla |
| Miguel Martínez Marín | Hospital Royo Villanova |
| Miriam Sandín Rollán | H.G.U. Alicante |
| Miryam Martínez Pascual del Riquelme | Hospital Universitario Los Arcos del Mar Menor |
| Mónica Delgado Ortega | Hospital Reina Sofía Cordoba |
| Nieves-M Oller Varela | Cl. S. Jordi |
| Nuria Farre Lopez | Hospital del Mar |
| Nuria Vallejo Camazon | H.Germans TRias i PUjol |
| Olga Guri Baiget | Hospital Moises Broggi |
| Pablo García García | Hospital Don Benito Villanueva |
| Pablo Santiago Díaz | H.A.R. LOJA |
| Patricia Couto | Hospital Universitario Nuestra Señora de Candelaria |
| Pedro Cabeza Lainez | Hospital Universitario Puerta del Mar, Cádiz |
| Pedro Casas Giménez | Hospital Valle de los Pedroches |
| Pedro José Flores Blanco | Hospital Lorenzo Guirao, Cieza, Murcia |
| Pedro Mª García Urruticoechea | H.G.U. Santa Lucia Cartagena |
| Pepa Sánchez Borque | Fundación Jiménez Díaz |
| Rafael Raso Raso | Hospital de Alcoy |
| Ramón De Castro Ariméndiz | Hospital Universitario de Tarragona Joan XXIII |
| Raquel Ancin Viguiristi | Complejo Hospitalario de Navarra |
| Regina Dalmau González-Gallarza | Hospital Universitario la Paz |
| Ricardo Pavón Jiménez | Hospital de Valme |
| Roberto Matía Francés | Hospital Ramón y Cajal |
| Rocío Carda Barrio | Fundación Jiménez Díaz |
| Rosa Macías Ruiz | Hospital Universitario Virgen de las Nieves |
| Roser Casañas Muñoz | Hospital Sagrat Cor |
| Rubén García Martin | Hospital de Mendaro |
| Rubén Juárez Prera | Hospital Universitario de Canarias |
| Santiago Camacho Freire | Hospital Juan Ramón Jiménez |
| Santiago Heras Herreros | La Ribera |
| Sheila Casas Lago | Hospital da Costa |
| Vanessa Escolar Pérez | Hospital Universitario de Basurto |
| Verónica Quevedo Nelson | Hospital de Gran Canaria Dr. Negrin |
| Víctor Miguel Ortiz Martínez | Hospital de Sagunto |
| Zigor Madaria Marijuan | Hospital Universitario de Basurto |
|  |  |
