# Supplementary material for: A Delphi consensus on the management of oral anticoagulation in patients with non-valvular atrial fibrillation in Spain: ACOPREFERENCE study
Source: PLoS One. 2020 Jun 1;15(6):e0231565. doi: 10.1371/journal.pone.0231565 (PMC7263623; doi:10.1371/journal.pone.0231565)
Supplement: Attachment — Submitted filename: Response to reviewers.doc [file pone.0231565.s004.doc]

Dr. Carlos Escobar

Department of Cardiology

Hospital Universitario La Paz

Paseo de la Castellana, 261, 28046 Madrid, Spain

Tel.: +34 91 7277000

Fax: +34 91 7277050

E-mail: [escobar_cervantes_carlos@hotmail.com](mailto:escobar_cervantes_carlos@hotmail.com)

Madrid, February 14, 2020

Dear editor,

Please, find enclosed the revised version of our original manuscript "*A Delphi consensus on the management of oral anticoagulation in patients with non-valvular atrial fibrillation in Spain: ACOPREFERENCE study*” (Manuscript ID: PONE-D-19-21146).

We thank the academic editor and the two reviewers for their detailed and useful review of our manuscript. We appreciate the positive feedback, as well as the constructive suggestions to further improve our manuscript, and we are glad to answer to each of their valuable comments.

We have carefully revised the original manuscript and have modified it according to the editor´s and the reviewers’ comments. All the changes in the revised manuscript are tracked in order to be easily identified. In addition, detailed responses to all comments requested by the reviewers are provided below. We report the editor´s and reviewers’ concern in black, and our replies in blue.

We would be grateful if you reconsidered the revised manuscript for publication in *Plos One* after the revision requested by the editor and the reviewers.

All of the listed authors have read the revision and agree with the content of the revised version of the manuscript. I am the corresponding author, and I will be responsible for communicating with the other authors about revisions and final approval of the proofs.

We thank you for your kind consideration and we are looking forward to hearing from you.

Sincerely yours,

Dr. Carlos Escobar.

**Academic editor:**

The Delphi technique is used to systematically combine expert opinion in order to arrive at an informed group consensus on a complex problem.

Several studies in health education have used the traditional Delphi technique and the Delphi technique to determine consensus in a number of important need areas, as for example clinical governance.

In principle, the Delphi is a group method that is administered by a researcher or research team that assembles a panel of experts, poses questions, synthesizes feedback and guides the group towards common ground. The Delphi is a method for organizing conflicting values and experiences and facilitates the incorporation of multiple opinions into consensus. (41,42) This is achieved using iterative rounds of sequential surveys interspersed with controlled feedback reports and the interpretation of experts' opinion.

The ACOPREFERENCE project is an interesting nationwide Spanish multicentre 2-round Delphi study to 93 seek expert opinion on the management of anticoagulation therapy for patients with NVAF.

The manuscript has important limitations in the results presentation:

1) The panel of selected healthcare experts is crucial. In the Study Design (Delphi Process) and in the Results (Characteristic of panel expert) the authors should add: %academic hospitals / number years of tenure in this academic hospital

**Response:** We appreciate the editor´s suggestion in order to improve the characterization of the Delphi expert panel. Therefore, according to the editor´s comment, we have now included in the manuscript the information regarding the percentage of Delphi expert panellists working in a University Hospital. This information has been included as a part of the characteristics of the Delphi expert panel displayed in Table 1 in the Results section, page 8. However, the information regarding the number years of tenure in these University Hospitals was not collected at the time when the study was conducted. Nevertheless, we consider that the lack of this information is not a limitation for the characterization of the experts who were selected based on their experience in and knowledge of the management of patients with non-valvular atrial fibrillation (NVAF) and anticoagulation regardless of the type of hospital where they were treating these patients. Nevertheless, we agree with the editor that this information would be interesting to complete the characterization of the experts and therefore, we will take it into account for future studies.

2) In the Results and in the tables the different response rate in round 1 /2/3 are not clearly expressed (histograms could be very helpful) consequently the discussion is limited by these important two variables (selection of experts and response rate). I'd like to review the manuscript after these important revisions in the Results.

**Response:** We appreciate the editor´s valuable recommendation in order to improve the exposure of the results of the 2-step Delphi process. We are pleased to have the opportunity to improve this issue so that the results obtained in this study are clearly shown in the manuscript. According to the helpful suggestion, we have now redesigned the exposure of the data obtained from the Delphi process. For this purpose, we have removed Tables 2-5 included in the Results section of the original manuscript (lines 237-243) and we have now included 4 figures instead (Figures 2-5) where the results from round 1 and round 2 of the Delphi process has been detailed. Tables 2-5 initially included in the manuscript only showed the percentage of experts scoring 7 to 9 (accepted) and the result after 2 rounds (agreed in round 1, agreed in round 2, etc.) for each item. However, we have now included 4 Figures where the percentage of patients scoring 1-3 (item rejected), 4-6 (undetermined), and 7-9 (accepted) in round 1 and round 2, respectively, are shown. Moreover, according to the reviewer´s comment (reviewer 2) we have also reduced the length of the items and used simpler phrases for each item. Consequently, we have now provided an appendix (S1 Appendix) including the complete description of the items of the 4 dimensions (Evaluation of thromboembolic and bleeding risk for treatment decision-making: 18 items; Choice of anticoagulant treatment for patients with NVAF: 39 items; Patient participation and education: 42 items; Use of anticoagulants in specific cardiology situations: 12 items).

The captions of these new Figures are included in the Results section, pages 10-12, lines 231-298:

- Fig 2. Results of the two-step Delphi process for the items relating to the evaluation of thromboembolic and bleeding risk for treatment decision
- Fig 3. Results of the two-step Delphi process for the items relating to the choice of anticoagulant treatment in patients with NVAF
- Fig 4. Results of the two-step Delphi process for the items relating to the participation and education of the anticoagulated patient
- Fig 5. Results of the two-step Delphi process for the items relating to the use of anticoagulants in specific cardiology situations

**Reviewer 1:**

Thank you for the invitation to review this manuscript, in this interesting paper, the authors described a Delphi consensus on the management of oral anticoagulation in patients with non-valvular atrial fibrillation in Spain, the article is in the well written and sound, however, some of the weakness bring to attention, please re-design or re-organize the tables, most of them are way too busy, description is too long and easy to lost, please try to use simple words or phrases to take place.

**Response:** We thank the reviewer for the positive feed-back and for his/her suggestion to redesign the exposure of the results of the 2-step Delphi process in the manuscript. We agree with the reviewer that Tables 2-5 included in the Results section of the original manuscript (lines 237-243) are too busy given that the description of the original items from the Delphi questionnaire was generally quite long. According to the helpful suggestion, we have redesigned the exposure of the data obtained from the two-step Delphi process in order that the results obtained in round 1 and round 2 are clearly shown in the manuscript. For this purpose, we have removed Tables 2-5 and we have now included 4 figures instead (Figures 2-5) where the results from round 1 and round 2 of the Delphi process has been detailed. These figures show the percentage of patients scoring 1-3 (item rejected), 4-6 (undetermined), and 7-9 (accepted) in round 1 and round 2, respectively. Moreover, according to the reviewer´s valuable recommendation we have also reduced the length of the items and used simpler phrases for each item.

The captions of these new Figures are included in the Results section, pages 10-12, lines 231-298:

- Fig 2. Results of the two-step Delphi process for the items relating to the evaluation of thromboembolic and bleeding risk for treatment decision
- Fig 3. Results of the two-step Delphi process for the items relating to the choice of anticoagulant treatment in patients with NVAF
- Fig 4. Results of the two-step Delphi process for the items relating to the participation and education of the anticoagulated patient
- Fig 5. Results of the two-step Delphi process for the items relating to the use of anticoagulants in specific cardiology situations

**Reviewer 2:**

Just like the authors mentioned, the major concern is the bias from the selection of experts and the subjectivity linked to the potentially divergent personal opinions of the panellists which may partly result from unevenly distributed expertise. Overseas experts might help with the regional biased opinion.

**Response:** We appreciate the reviewer´s comment regarding the Delphi expert panellists. As we have mentioned in the Discussion section of the manuscript, we must assume the obvious concerns with regard to a Delphi panel study such as the potential bias derived from the selection of experts and the subjectivity linked to the potentially divergent opinions of the panellists which may partly result from unevenly distributed expertise. However, although the participant experts may have different level of expertise, all of them were selected based on their extensive experience in and knowledge of the management of patients NVAF and anticoagulation. In addition, we consider that the participation of a large number of experts from hospitals distributed homogeneously throughout Spain may avoid the regional biased opinion in this national study given the potential differences in the management of oral anticoagulation (OAC) between the different Spanish regions (Autonomous Communities in Spain) due to administrative issues. Furthermore, although the participation of overseas experts may be interesting in other studies with a different purpose, this was a national study aiming to achieve consensus among Spanish cardiologists on the selection and management of OAC in patients with NVAF.

**Other changes**

As a consequence of the elimination of Tables 2-5 in the revised version of the manuscript, the previous Table 6 (Expert-based Delphi consensus recommendations for the management of oral anticoagulation in patients with NVAF) is now Table 2 in the current version of the manuscript.

**Journal requirements**

2) Please clarify whether the panel members provided their written informed consent to participate in this study.

**Response:**

The Delphi panel members were invited to participate in the study using an informative document outlining the aims and the study procedure. After being formally invited and informed, all experts who wanted to participate in the study as members of the Delphi expert panel had to sign a contract.
